# Case study: positive outcomes from a negative

**DOI:** 10.1186/1897-4287-10-S2-A57

**Published:** 2012-04-12

**Authors:** R Williams, K Tucker

**Affiliations:** 1 Hereditary Cancer Clinic, Prince of Wales Hospital, Randwick NSW 2031, Australia

## Background

As the work load for clinical genetics escalates and more genetic test are ordered, the potential for errors increase. This report present at the affected patient’s request, the occurrence of an error and its subsequent management.

A BRCA2 large deletion had been detected in a family member and predictive testing had already occurred in other family members. Our client had 50/50 chance of having the mutation and had a negative predictive test. When breast cancer occurred in the patient, the sample was retested and found to be positive. The different results were given in person and a root cause analysis done. The patient requested that the error be discussed at clinical meetings so lessons found could be learnt by the whole genetics community.

Multiple factors impacted on this case. An unclassified variant-considered likely to be benign, had been identified, as well as a pathogenic mutation. Blood was collected from both relatives one day apart and sent for testing at the laboratory which identified the mutation. Although the sample request form requested a predictive test specifying the gene, lab ID and DOB of the proband it did not specify the mutation to avoid transcription errors. A new laboratory staff member incorrectly tested for the unclassified variant. Although duplicate testing was done the samples were not collected independently and the same error occurred. The clinical staff were rushed as there were 2 carriers in the breast clinic, one a newly diagnosed breast cancer and another a possible diagnosis, requesting consultations.

A comprehensive review of the clinic’s genetic testing protocol has tightened protocol to minimize future error; the request form is accompanied by a de-identified copy of the mutation even if the testing laboratory did the mutation search. Although the protocol required predictive test results to be checked against the proband’s result results will now be signed by the doctor and counselor before being given. Cost consideration are ongoing as to whether the second sample will be sent completely separately. Efforts are being made to prevent the clash with the breast clinic.

Errors in genetic testing are rare event and give the opportunity to review procedures. Knowledge of errors allow other clinicians to review their protocols. We have learnt not only from our errors but from the valuable input from other clinicians who have shared their traumatic experiences. We propose that documentation of the extent and cause of genetic testing errors occur at the family cancer clinic day next year or at the COSA meeting. A culture of open disclosure with colleagues as well as the clients affected will help guard against further avoidable errors and help us develop sustainable, attainable and cost effective processes.

